# Tobacco Use among HIV-Positive and HIV-Negative Women and Men in Zambia—Demographic and Health Survey, 2018

**DOI:** 10.3390/ijerph19073859

**Published:** 2022-03-24

**Authors:** Alissa C. Kress, Carlen Stadnik, Masauso Moses Phiri, Fastone M. Goma, Evelyn Twentyman

**Affiliations:** 1 Office on Smoking and Health, National Center for Chronic Disease Prevention and Health Promotion, Centers for Disease Control and Prevention, Atlanta, GA 30341, USA; ydj2@cdc.gov; 2 Oak Ridge Institute for Science and Education, Oak Ridge, TN 37830, USA; pmu8@cdc.gov; 3 Center for Primary Care Research, School of Medicine, University of Zambia, Lusaka 10101, Zambia; masauso@unza.zm; 4 Centre for Primary Care Research, Lusaka 10101, Zambia; gomafm@yahoo.co.uk

**Keywords:** HIV, tobacco use, tobacco smoking, Zambia

## Abstract

Country-specific estimates of tobacco use among people living with HIV (PLWH) are lacking for much of sub-Saharan Africa. We aim to evaluate the association between the HIV status and tobacco product use status, frequency, and intensity, using nationally representative data from Zambia. We analyzed data from the 2018 Demographic and Health Survey conducted in Zambia among women aged 15–49 years and men aged 15–59 years. We performed logistic regression to assess the associations of HIV status, selected sociodemographic, and other characteristics with indicators of tobacco use (i.e., status, frequency, and intensity). Among women, 14.3% tested positive for HIV and 2.7% reported current smoking or tobacco use; women living with HIV were more likely to report currently smoking or using tobacco than women living without HIV (4.4% vs. 2.4%; aPR: 1.46). Among men, 8.4% tested positive for HIV and 19.5% reported current smoking; men living with HIV were more likely than men living without HIV to report current smoking (27.8% vs. 18.7%; aPR: 1.22). Several sociodemographic characteristics were associated with tobacco use, including age, residence (urban/rural), education level, employment status, and wealth index. The frequency and intensity of smoking among men who currently smoked did not differ by HIV status. Tobacco use was more likely in PLWH than those without HIV in Zambia. Our findings highlight the need to encourage and support tobacco cessation among PLWH, possibly by offering cessation services at existing intersections with health care or integrating cessation support into mHealth and other alternative models of care.

## 1. Introduction

The global burdens of both HIV and tobacco use are staggering; in 2019, over 1.1 billion people smoked tobacco and 38 million people were living with HIV [1,2]. People living with HIV (PLWH) are more likely to smoke cigarettes and use other types of tobacco compared to people living without HIV (PLWOH) [3,4,5], with a recent meta-analysis finding that nearly half of PLWH were current smokers and nearly one-quarter were former smokers [6]. Smoking is particularly hazardous for PLWH, as it increases the risk for developing HIV-related infections, cardiovascular disease (CVD), respiratory diseases, and cancers [7,8,9]. Where antiretroviral therapy (ART) for HIV is available, tobacco smoking results in a greater loss of life years than the HIV infection itself among PLWH who smoke [10]. The co-occurrence of HIV alongside noncommunicable diseases (NCDs) and their risk factors, such as tobacco use, has been described as a joint or synergistic epidemic [11,12].

More than two-thirds of all PLWH reside in sub-Saharan Africa (SSA), a region of the world where tobacco use has been steadily increasing [2,13]. Recent multicountry studies of tobacco use and HIV have been published using Demographic and Health Survey (DHS) data [3,4]. Murphy et al. (2019) analyzed DHS data from 25 countries in SSA and found PLWH were more likely to smoke cigarettes and use smokeless tobacco (SLT) than PLWOH [3]. Similarly, Mdege et al. (2017) analyzed DHS data from 28 low- and middle-income countries (LMIC), many of which were in SSA, and found a higher prevalence of any tobacco use and tobacco smoking among men living with HIV (MLWH) compared to men living without HIV (MLWOH); for women, they also noted a higher prevalence of SLT use among those with HIV [4]. The global and multicountry estimates described here clearly indicate an urgent need to better characterize country-specific estimates for tobacco use, NCDs, and related risk factors among PLWH. Unfortunately, these country-specific estimates are lacking for much of SSA, and for LMICs in particular [11,14,15].

The focus of our current study is Zambia, an LMIC in SSA with one of the largest populations of PLWH [16], where tobacco use has been steady over time [17,18], and with recent (2018) DHS data available on tobacco use and HIV. In Zambia, approximately 1% of women and nearly 20% of men report smoking tobacco, while about 14% of women and 7.5% of men are HIV-positive [18]. Both of the aforementioned multicountry studies included 2013–2014 data from Zambia [3,4]; however, HIV prevalence in Zambia has declined since then [3,4,18] and neither study assessed the relationship between HIV status and the frequency or intensity of tobacco use. Therefore, we aim to update previous estimates using more recent data from 2018, as well as evaluate the association between HIV status and the status, frequency, and intensity of tobacco use using nationally representative data from Zambia.

## 2. Materials and Methods

### 2.1. Data Source

We performed secondary data analyses on data from the 2018 DHS conducted in Zambia. DHS are nationally representative surveys that collect data on a variety of demographic, health, fertility, and nutrition topics. The survey is conducted among women aged 15–49 years and men aged 15–59 years [19]. 

The 2018 Zambia DHS (ZDHS) comprised four components: household questionnaire, woman’s questionnaire, man’s questionnaire, and biomarker questionnaire. The household questionnaire collected demographic information on members and visitors of each household, as well as household characteristics. The woman’s and man’s questionnaires collected information on demographic characteristics, knowledge and behavior related to health issues such as tobacco use, HIV and STIs, and other topics. The woman’s questionnaire collected additional information on maternal and reproductive health, maternal and child nutrition, and domestic violence. The biomarker questionnaire recorded height, weight, hemoglobin, and HIV test results [18]. 

The 2018 ZDHS followed a stratified two-stage sample design, consistent with standard DHS practice [19]. First clusters were selected within sampling stratum; then, households were sampled within clusters. Of the 12,943 occupied households sampled, 12,831 were interviewed, resulting in a household response rate of 99%. Women aged 15–49 years and men aged 15–59 years who were permanent residents or stayed in the household the night preceding the survey were eligible to be interviewed. Of the 14,189 women eligible, 13,683 were interviewed (response rate of 96%); of the 13,251 men eligible, 12,132 were interviewed (response rate of 92%) [18].

### 2.2. HIV Status

HIV status was based on results of blood tests conducted as part of the biomarker questionnaire. We categorized HIV status as HIV-positive, which included those who tested positive for either HIV-1 or HIV-2, and HIV-negative. Consistent with a previous report, those whose test results were inconclusive were considered missing [20].

### 2.3. Tobacco Use

Women who reported they currently smoked cigarettes or currently smoked or used any other type of tobacco every day or some days at the time of the survey were considered to currently use any tobacco (Appendix A). Because 72.6% of women who currently smoked cigarettes also used other types of tobacco, we opted not to examine these categories separately.

We created three variables to assess smoked tobacco use among men: tobacco smoking status, frequency of smoked tobacco use, and intensity of smoked tobacco use (Appendix B). For tobacco smoking status, men who reported they currently smoked tobacco every day or some days were considered to currently smoke tobacco, those who smoked tobacco every day or some days in the past but not currently were considered to have formerly smoked tobacco, and those who did not smoke tobacco currently or in the past were considered to have never smoked tobacco. We created a frequency of smoked tobacco use variable based on corresponding responses to current tobacco smoking (every day, some days, or not at all). We also calculated an intensity of smoked tobacco use variable by summing daily use of each tobacco product used and categorizing those sums as 5 or more times per day, 1–4 times per day, and <1 time per day. Some days, users were considered to use tobacco products <1 time per day, and nonusers were categorized as “do not smoke tobacco”. Every day, users who reported “less than daily” or “less than weekly” use of tobacco products were considered missing, resulting in some respondents appearing as daily tobacco smokers but missing data on intensity of smoked tobacco use.

Similar questions concerning use of SLT were also asked of men. However, due to low prevalence (1.0%) of SLT use among men, separate analyses of SLT use as an outcome were not possible. Instead, we included use of SLT (yes, no) as a covariate for analyses involving men.

### 2.4. Covariates

We included covariates age group (15–24 years, 25–34 years, 35–49 years, 50–59 years (men only)), residence (urban, rural), highest education level attended or completed (no education, primary, secondary or higher), employment status (currently employed, not currently employed), wealth index (lowest, lower, middle, higher, highest), and recent sexual activity (not sexually active in last 4 weeks, sexually active in last 4 weeks). To determine wealth index, households were given scores based on owned consumer goods and housing characteristics. Each household member was assigned the household score, and each person was ranked by their score. The distribution was then divided into five equal categories, or quintiles [18].

For women, we also included problems accessing health care (yes, no) and pregnancy status (currently pregnant, not currently pregnant or not sure). Women who identified at least one of the following as a big problem when sick and wanting to get medical advice or treatment were considered to have problems accessing care: getting permission to go for treatment, distance to the health facility, not wanting to go alone, having to take transport, concern that there may not be any health provider, concern that there may not be a female health provider, or rude attitude of health provider.

### 2.5. Statistical Analyses

For this study, we combined the 2018 woman’s, man’s, and biomarker data files, then applied weights provided with the biomarker file to calculate appropriate nationally representative estimates for HIV. We calculated descriptive statistics, including percentages and corresponding 95% confidence intervals (95% CI), for selected sociodemographic and other characteristics and indicators of tobacco use stratified by HIV status. We conducted chi-square analyses to test for significant differences in the distribution of these characteristics by HIV status; a *p*-value of <0.05 was considered statistically significant. We performed binary or ordinal logistic regression, as appropriate, to assess the associations of HIV status and selected sociodemographic and other characteristics with indicators of tobacco use. We used the ADJRR option on the PREDMARG statement to calculate model-adjusted prevalence ratios (PR) and 95% CIs [21]. All analyses were conducted separately for men and women. Analyses were conducted using SAS v9.4 and SAS-callable SUDAAN v11.0.

Of the initial 25,815 women and men interviewed, we excluded 1264 (4.9% (624 women and 640 men)) who either did not participate in the HIV testing portion of the survey or whose test results were inconclusive. We further excluded 30 men who reported smoking tobacco every day or some days but also indicated they did not use any of the individual smoked tobacco products. Our resulting analytic sample consisted of 13,059 women and 11,462 men.

## 3. Results

Overall, 14.3% of women tested HIV-positive (Table 1). Most women living with HIV (WLWH) were 35–49 years of age (47.0%), whereas most women living without HIV (WLWOH) were 15–24 years of age (46.5%). A higher proportion of WLWH resided in urban areas (66.6% vs. 43.1%), attended or completed secondary education or higher (51.3% vs. 47.3%), and were currently employed (56.8% vs. 42.9%) compared with WLWOH. A smaller proportion of WLWH had problems accessing healthcare and were currently pregnant than WLWOH (46.1% vs. 52.1% and 6.0% vs. 8.6%, respectively). Among WLWH, the proportion belonging to each wealth index quintile ranged from 8.4% in the lowest quintile to 33.8% in the higher quintile; for WLWOH, the proportion ranged from 18.4% in the middle quintile to 23.2% in the highest quintile. Over half of all women (53.0%) reported being sexually active in the last four weeks, with no difference between WLWH and WLWOH. Overall, 2.7% of women reported current tobacco use, with a higher prevalence among WLWH than WLWOH (4.4% vs. 2.4%). 

Overall, 8.4% of men tested HIV-positive (Table 2). Most MLWH were between 35 and 49 years of age (49.8%), whereas most MLWOH were 15–24 years of age (43.1%); nearly one in five (17.0%) MLWH were 50–59 years of age, while only 7.1% of MLWOH were in this age group. Compared to MLWOH, more MLWH resided in urban areas (60.7% vs. 42.5%), attended or completed secondary education or higher (61.7% vs. 56.4%), were currently employed (88.0% vs. 74.0%), and had been sexually active in the last 4 weeks (72.1% vs. 57.0%). Among MLWH, the proportion belonging to each wealth index quintile ranged from 8.0% in the lowest quintile to 30.6% in the higher quintile; for MLWOH, the proportion ranged from 17.2% in the lowest quintile to 22.4% in the highest quintile. A small percentage (1.0%) of all men reported using SLT. Over one-quarter (27.8%) of MLWH reported they currently smoked tobacco and 12.2% formerly smoked tobacco, compared with 18.7% and 7.4%, respectively, of MLWOH. A higher proportion of MLWH reported currently smoking every day and some days than MLWOH (21.4% vs. 14.2% and 6.4% vs. 4.6%, respectively). Compared to MLWOH, more MLWH smoked tobacco 5 or more times per day (10.7% vs. 7.3%), 1–4 times per day (9.0% vs. 6.8%), and <1 time per day (6.6% vs. 4.6%).

WLWH were more likely to currently use tobacco than WLWOH (aPR: 1.46) (Table 3). The prevalence of current tobacco use among women increased with an increasing age (0.9% (15–24 years) to 4.9% (35–49 years)). In general, the prevalence of current tobacco use among women decreased with an increasing education level (6.1% (no education) to 1.8% (secondary or higher)) and wealth index (4.4% (lowest) to 1.7% (highest)). After adjustment for covariates, the prevalence of current tobacco use was significantly higher among women in higher age groups (aPRs: 3.01 (25–34 years), 4.16 (35–49 years)), who resided in urban areas (aPR: 1.78), had no education (aPR: 1.79), and were in the lowest to middle wealth index quintiles (aPRs ranged from 3.87 (lowest) to 1.83 (middle)), compared to those who were 15–24 years of age, resided in rural areas, had completed secondary or higher education, and were in the highest wealth index quintile. 

MLWH were more likely than MLWOH to report current or former tobacco smoking (aPR: 1.22 and 1.12, respectively) (Table 4). The prevalence of current tobacco smoking was 27.8% among MLWH, 31.6% among men aged 50–59 years, 21.1% among men residing in rural areas, 27.6% among men with no education, 30.1% among those in the lowest wealth index quintile, and 70.4% among men currently using SLT. After adjusting for covariates, the prevalence of current smoking was significantly higher among MLWH (aPR: 1.22), men residing in urban areas (aPR: 1.63), with no or primary education (aPRs: 1.26 and 1.25, respectively), who were currently employed (aPR: 1.32), and who currently used SLT (aPR: 3.54), compared to MLWOH, men residing in rural areas, who had completed secondary or higher education, who were not currently employed, and who did not currently use SLT. The prevalence of current tobacco smoking increased with the increasing age group (aPRs ranged from 2.17 (25–34 years) to 2.76 (50–59 years)) and decreasing wealth index quintile (aPRs ranged from 1.28 (higher) to 2.62 (lowest)) compared to men 15–24 years of age and in the highest wealth index quintile. The prevalence of former tobacco smoking was 12.2% among MLWH, 10.7% among men aged 50–59 years, and 8.2% among men with secondary or higher education. After adjusting for covariates, the prevalence of former tobacco smoking was significantly higher among MLWH (aPR: 1.12), men residing in urban areas (aPR: 1.32), with no or primary education (aPRs: 1.15 and 1.15, respectively), and who were currently employed (aPR: 1.19), compared to MLWOH, men residing in rural areas, who had completed secondary or higher education, and who were not currently employed. The prevalence of former tobacco smoking increased with an increasing age (aPRs ranged from 1.73 (25–34 years) to 1.95 (50–59 years)) and decreasing wealth index (aPRs ranged from 1.20 (higher) to 1.77 (lowest)), compared to men 15–24 years of age and those in the highest wealth index quintile.

Among men who currently smoked, the frequency of smoked tobacco use varied by selected characteristics. MLWH had a similar prevalence of smoking tobacco every day and some days as MLWOH (76.8% vs. 75.6% and 23.2% vs. 24.4%, respectively) (Table 4). The prevalence of every day smoking was 84.6% among men aged 50–59 years, 81.9% among those with primary education, and 82.6% among those in the lower wealth index quintile. After adjusting for covariates, the prevalence of every day smoking was significantly higher among men in higher age groups (aPRs: 1.19 (35–49 years), 1.21 (50–59 years)) and who had completed primary education (aPR: 1.16), compared with those aged 15–24 years and who had completed secondary or higher education. The prevalence of some days smoking was 36.3% among those 15–24 years of age and 33.5% among those with secondary or higher education. The prevalence of some days smoking was significantly lower among men in higher age groups (aPRs: 0.60 (35–49 years), 0.56 (50–59 years)) and who had completed primary education (aPR: 0.64). 

Among men who currently smoked, the intensity of smoked tobacco use varied by selected characteristics. The prevalence of smoking tobacco ≥5 times per day (40.8% vs. 39.3%), 1–4 times per day (34.3% vs. 36.2%), and <1 time per day (25.0% vs. 24.6%) was similar among MLWH and MLWOH (Table 4). The prevalence of smoking tobacco ≥5 times per day increased with an increasing age (range: 26.2% (15–24 years) to 47.4% (50–59 years)); conversely, smoking tobacco <1 time per day decreased with an increasing age (37.1% (15–24 years) to 15.6% (50–59 years)). Approximately one-third of men who completed secondary or higher education used smoked tobacco ≥5 times per day, 1–4 times per day, and <1 time per day (35.8%, 30.3%, and 33.8%, respectively). After adjusting for covariates, the prevalence of smoking tobacco ≥5 times per day was significantly higher among men in higher age groups (aPRs: 1.33 (25–34 years), 1.71 (35–49 years), 1.75 (50–59 years)) and among those who had completed primary education (aPR: 1.26), compared with those aged 15–24 years and who had completed secondary or higher education. Men aged 35–49 years (aPR: 0.93) and with primary education (aPR: 0.95) were less likely to use smoked tobacco 1–4 times per day; men in higher age groups (aPR: 0.76 (25–34 years), 0.55 (35–49 years), 0.53 (50–59 years)) and with primary education (aPR: 0.75) were also less likely to use smoked tobacco <1 time per day.

## 4. Discussion

We found that tobacco use and tobacco smoking were more likely among WLWH and MLWH, respectively, than PLWOH in Zambia; however, the frequency and intensity of tobacco smoking among men who currently smoked did not differ by HIV status. Several sociodemographic factors were also associated with tobacco use in these populations. Information gleaned from this study can help inform the development of programs and policies that encourage and support tobacco use cessation among PLWH in Zambia.

We found that HIV was significantly associated with current tobacco use in women and current tobacco smoking in men, a finding which aligned with previously published literature [3,4,5,6]. However, among men who currently smoked tobacco, HIV was not associated with either the frequency or intensity of smoked tobacco use (we were unable to assess these measures for women). It is possible that the sample sizes in these categories were too small to detect significant differences; however, aPRs for these associations were all near the null value of 1.0. Alternatively, the lack of association noted in these measures may indicate that current smokers living with and without HIV are not significantly different in terms of the frequency and intensity of tobacco smoking. Few studies have assessed the frequency or intensity of tobacco smoking among PLWH who smoke; Asfar et al. (2021) found that in the US, the number of cigarettes smoked per day by PLWH was lower than by PLWOH (9.9 vs. 18.2, respectively) [5], while Mdege et al. (2021) found that PLWH in Uganda reported an average of 5.2 manufactured cigarettes smoked per day [22]. It is known that the number of cigarettes smoked per day does not fully capture the intensity of smoking, given interindividual differences in the depth of smoke inhalation and related measures [23]. A higher intensity of smoking has, however, been linked to higher levels of inflammatory biomarkers, such as C-reactive protein, which in turn is associated with an increased risk of morbidity and mortality among PLWH [24]; therefore, PLWH who smoke tobacco may benefit from support to reduce the frequency or intensity of smoking until they are able to quit smoking altogether. 

We identified several sociodemographic characteristics that were associated with tobacco use among women and tobacco smoking among men, including age, residence (urban/rural), education level, employment status, and wealth index. Our findings were consistent with previous studies which also demonstrated that those in older age groups [25,26,27], with lower levels of education [3,25,28,29,30,31,32], employment such as household service, manual, or agricultural work [3,30,31], or lower wealth [3,32] were more likely to use tobacco. Perhaps paradoxically, we found that tobacco use was more prevalent among residents of rural areas; however, once we controlled for other sociodemographic characteristics in logistic regression models, residents of urban areas were more likely to use or smoke tobacco. Prior studies have noted a higher smoking prevalence in rural areas of Zambia; nearly 40% in males and 10% in females in the rural districts of Kaoma and Kasama [33], and approximately 18% in males and 2% in females in the urban district of Lusaka [34]. Other studies conducted using DHS data from SSA, which included data from Zambia, have found urban residents more likely to use or smoke tobacco [30,31]. This discrepancy could be explained, in part, by underlying differences in characteristics of urban and rural residents in Zambia such as age and education level [33,34], or economic factors that may influence the access to and availability of tobacco products [35]. Among men who currently smoked, older age and a lower education level were also associated with smoking tobacco every day and smoking tobacco five or more times per day. Previous literature has shown mixed results for predictors of smoking intensity, depending on whether measures were categorical or continuous, and further study is warranted [30,31]. Finally, although a prior study found an association between recent sexual activity and tobacco use [3], ours did not; future research may be needed to elucidate this relationship.

The findings of this study are relevant to ongoing efforts in SSA and around the world to integrate cessation support into health systems serving PLWH. Though not significant in logistic regression models, WLWH less frequently reported problems accessing health care than WLWOH. This might present an opportunity to promote cessation through existing healthcare visits, and to continue to expand access to health care for all women. This frequency of healthcare system engagement among PLWH has previously been identified as a potential facilitator of cessation support for PLWH [36]. Additionally, some previous studies suggest PLWH may be more inclined to quit smoking after receiving their HIV diagnosis, enrolling in an HIV clinic, or initiating ART [28,37]. Kintu et al. (2020) recommended gradually integrating NCD care, including tobacco use screening and counseling, into existing HIV services in SSA [14]. The Zambia Consolidated Guidelines for the Treatment and Prevention of HIV Infection (2020) offers smoking cessation as a possible intervention to prevent and manage CVD, and recommends counseling for “positive living” at every routine visit for PLWH, including “nutrition, alcohol, and smoking cessation” [38]. To our knowledge, no study has evaluated these recommendations; however, studies of smoking cessation interventions in HIV clinics in LMICs are underway [39,40]. Applicable lessons learned for the integration of tobacco cessation counseling into routine clinical care for PLWH may also be available within the experience of cessation integration in care for people with tuberculosis [41]. It may also be possible to implement integrated care along with broader health system reforms which expand care to the general population, increase the use of mHealth technologies, or offer tobacco screening and cessation services in mobile clinics or home-based care [14,42]. Overcoming barriers to offering cessation for PLWH wherever possible is imperative, as even nonsustained periods of abstinence have proven benefits [36]. 

This study was subject to several limitations. First, except for data on HIV status, data included in this study were self-reported by participants, which could result in social desirability or recall biases. Second, data gleaned from DHS were cross-sectional in nature and, therefore, causality could not be determined. Third, data were not available for several factors, which may be related to either HIV or tobacco use, including ART [28], mental health [5,22,25,32], and substance use [5,22,28,30,32]; thus, we were unable to account for these factors. Additional health indicators, such as domestic violence or maternal health, may also be associated with HIV or tobacco use; the inclusion of these indicators was beyond the scope of the current study but may warrant future exploration. We were also unable to fully assess SLT use due to small sample sizes, though we did control for it in analyses among men. Fourth, due to the low prevalence of tobacco use reported, we were unable to assess cigarette smoking and other types of tobacco use separately among women. This may also have resulted in weaker associations for some of the factors we assessed in women; this was not unexpected, as tobacco use has consistently been found to be lower in women than men in SSA [4,25,26,43]. Finally, due to the design of DHS, we included limited ages in this study; there were, therefore, inherent limitations in our ability to depict the relationship between age and tobacco use, particularly for older adults. Despite these limitations, to our knowledge, this was the first study using nationally representative DHS data to evaluate the relationship between HIV and other factors with tobacco use in women and men in Zambia.

## 5. Conclusions

Tobacco use and tobacco smoking were more likely in WLWH and MLWH, respectively, than PLWOH in Zambia; however, among men who currently smoked tobacco, the frequency and intensity of smoking did not differ by HIV status. Several other sociodemographic factors were also associated with tobacco use in these populations. Our findings highlight the importance of encouraging and supporting tobacco cessation among PLWH, possibly by offering cessation services at existing intersections with health care, tailoring cessation interventions and campaigns separately to rural and urban audiences, and integrating cessation support into mHealth and other alternative models of care. Additionally, given that smoking is on the rise among women [43] and women account for a large proportion of new HIV infections in SSA [2], additional efforts to curb tobacco use in WLWH may be warranted.

## Figures and Tables

**Table 1:** Distribution of selected sociodemographic and other characteristics, and tobacco use, among women aged 15–49 years who were HIV-positive and HIV-negative—2018 DHS, Zambia.

	Overall (*n* = 13,059)	HIV-Positive (*n* = 1711)	HIV-Negative (*n* = 11,348)
	% (95% CI)	% (95% CI)	% (95% CI)
Total	N/A	14.3% (13.3–15.5%)	85.7% (84.5–86.8%)
Age group ^1^			
15–24 years	42.2% (41.3–43.1%)	16.6% (14.0–19.4%)	46.5% (45.5–47.5%)
25–34 years	29.9% (28.9–30.8%)	36.5% (33.9–39.2%)	28.8%(27.8–29.8%)
35–49 years	28.0% (27.1–28.8%)	47.0% (43.7–50.3%)	24.8% (23.9–25.7%)
Residence ^1^			
Urban	46.5% (43.7–49.3%)	66.6% (62.1–70.8%)	43.1% (40.5–45.8%)
Rural	53.5%(50.7–56.3%)	33.4% (29.2–37.9%)	56.9% (54.2–59.5%)
Highest education level attended or completed ^1^			
No education	7.6% (6.7–8.5%)	5.7% (4.5–7.4%)	7.9% (7.0–8.9%)
Primary	44.6% (42.7–46.5%)	42.9% (39.7–46.2%)	44.9% (43.0–46.8%)
Secondary or higher	47.8% (45.8–49.9%)	51.3%(47.9–54.8%)	47.3% (45.2–49.4%)
Employment status ^1^			
Currently employed	44.9% (43.4–46.4%)	56.8% (53.3–60.3%)	42.9% (41.3–44.4%)
Not currently employed	55.1% (53.6–56.6%)	43.2% (39.7–46.7%)	57.1% (55.6–58.7%)
Wealth index ^1^			
Lowest	17.8% (16.4–19.3%)	8.4% (6.9–10.3%)	19.3% (17.9–20.9%)
Lower	17.6% (16.2–19.1%)	11.6% (9.7–16.7%)	18.6% (17.2–20.1%)
Middle	18.3% (16.8–19.9%)	17.2% (14.3–20.6%)	18.4% (16.9–20.1%)
Higher	22.4% (19.7–25.3%)	33.8% (27.2–41.0%)	20.5% (18.2–22.9%)
Highest	24.0% (21.8–27.1%)	29.1% (24.1–34.6%)	23.2% (20.5–26.0%)
Recent sexual activity			
Sexually active in last 4 weeks	53.0% (51.6–54.4%)	55.4% (51.4–59.3%)	52.6% (51.2–54.0%)
Not sexually active in last 4 weeks	47.0% (45.6–48.4%)	44.6% (40.7–48.6%)	47.4% (46.0–48.8%)
Problems accessing health care ^1^			
Yes	51.2% (49.4–53.0%)	46.1% (43.0–49.2%)	52.1% (50.1–54.0%)
No	48.8% (47.0–50.6%)	53.9% (50.8–57.0%)	47.9% (46.0–49.9%)
Pregnancy status ^1^			
Currently pregnant	8.2% (7.7–8.8%)	6.0% (4.8–7.5%)	8.6% (7.9–9.4%)
Not currently pregnant or not sure	91.8% (91.2–92.3%)	94.0% (92.5–95.2%)	91.4% (90.6–92.1%)
Currently use any tobacco ^1^			
Yes	2.7% (2.4–3.1%)	4.4% (3.5–5.6%)	2.4% (2.1–2.8%)
No	97.3% (96.9–97.7%)	95.6% (94.4–96.5%)	97.6% (97.2–97.9%)

Abbreviations: CI—confidence interval; DHS—Demographic and Health Survey. ^1^ Statistically significant difference (*p* < 0.05) in distribution of women living with HIV and women living without HIV, based on Chi-square test.

**Table 2:** Distribution of selected sociodemographic and other characteristics, and tobacco use, among men aged 15–59 who were HIV-positive and HIV-negative—2018 DHS, Zambia.

	Overall (*n* = 11,462)	HIV-Positive (*n* = 923)	HIV-Negative (*n* = 10,539)
	% (95% CI)	% (95% CI)	% (95% CI)
Total	n/a	8.4% (7.7–9.2%)	91.6% (90.1–92.3%)
Age group ^1^			
15–24 years	40.2% (39.2–41.2%)	8.5% (6.6–11.0%)	43.1% (41.9–44.3%)
25–34 years	25.6% (24.6–26.5%)	24.7% (21.7–28.1%)	25.7% (24.7–26.6%)
35–49 years	26.3% (25.2–27.4%)	49.8% (45.9–53.7%)	24.2% (23.0–25.3%)
50–59 years	7.9% (7.4–8.5%)	17.0% (14.3–20.0%)	7.1% (6.6–7.6%)
Residence ^1^			
Urban	44.0% (41.0–47.0%)	60.7% (55.6–65.4%)	42.5% (39.5–45.5%)
Rural	56.0% (53.0–59.0%)	39.4% (34.6–44.4%)	57.5% (54.5–60.5%)
Highest education level attended or completed ^1^			
No education	3.9% (3.4–4.6%)	2.9% (1.8–4.7%) ^2^	4.0% (3.4–4.7%)
Primary	39.2% (37.6–40.9%)	35.5% (31.6–39.5%)	39.5% (37.9–41.2%)
Secondary or higher	56.9% (55.1–58.6%)	61.7% (57.5–65.7%)	56.4% (54.6–58.2%)
Employment status ^1^			
Currently employed	75.2% (73.8–76.5%)	88.0% (84.8–90.7%)	74.0% (72.6–75.4%)
Not currently employed	24.8% (23.5–26.2%)	12.0% (9.4–15.2%)	26.0% (24.6–27.4%)
Wealth index ^1^			
Lowest	16.4% (15.1–17.9%)	8.0% (6.3–10.1%)	17.2% (15.8–18.7%)
Lower	17.9% (16.5–19.5%)	13.2% (10.9–15.9%)	18.4% (16.9–19.9%)
Middle	20.1% (18.4–21.8%)	18.5% (15.4–22.1%)	20.2% (18.6–22.0%)
Higher	22.6% (19.3–26.2%)	30.6% (24.5–37.5%)	21.9% (18.8–25.3%)
Highest	23.0% (20.1–26.2%)	29.7% (24.6–35.2%)	22.4% (19.6–25.5%)
Recent sexual activity ^1^			
Sexually active in last 4 weeks	58.2% (57.0–59.5%)	72.1% (68.3–75.6%)	57.0% (55.6–58.3%)
Not sexually active in last 4 weeks	41.8% (40.5–43.0%)	27.9% (24.4–31.8%)	43.1% (41.7–44.4%)
Current smokeless tobacco use			
Yes	1.0% (0.7–1.3%)	- ^3^	0.9% (0.7–1.2%)
No	99.1% (98.7–99.3%)	- ^3^	99.1% (98.8–99.4%)
Tobacco smoking status ^1^			
Currently smoke tobacco	19.5% (18.4–20.7%)	27.8% (24.6–31.3%)	18.7% (17.6–19.9%)
Formerly smoked tobacco	7.8% (7.1–8.6%)	12.2% (9.8–15.0%)	7.4% (6.7–8.2%)
Never smoked tobacco	72.7% (71.2–74.2%)	60.0% (55.9–64.0%)	73.9% (72.3–75.3%)
Frequency of smoked tobacco use ^1^			
Every day	14.8% (13.9–15.7%)	21.4% (18.4–24.6%)	14.2% (13.2–15.1%)
Some days	4.7% (4.2–5.3%)	6.4% (4.8–8.7%)	4.6% (4.1–5.2%)
Not at all	80.5% (79.3–81.6%)	72.2% (68.7–75.4%)	81.3% (80.1–82.4%)
Intensity of smoked tobacco use ^1^			
5 or more times per day	7.6% (6.9–8.4%)	10.7% (8.7–13.3%)	7.3% (6.7–8.1%)
1–4 times per day	6.9% (6.4–7.6%)	9.0% (7.1–11.4%)	6.8% (6.2–7.4%)
<1 time per day	4.8% (4.2–5.3%)	6.6% (4.9–8.8%)	4.6% (4.1–5.2%)
Do not smoke tobacco	80.7% (79.5–81.9%)	73.7% (69.9–77.1%)	81.4% (80.2–82.5%)

Abbreviations: CI—confidence interval; DHS—Demographic and Health Survey. ^1^ Statistically significant difference (*p* < 0.05) in distribution of men living with HIV and men living without HIV, based on Chi-square test. ^2^ Estimate based on 25–49 unweighted cases. ^3^ Data suppressed because the estimate was based on fewer than 25 unweighted cases.

**Table 3:** Prevalence and adjusted prevalence ratios (APRs) for HIV status and selected characteristics with any current tobacco use among women—2018 DHS, Zambia.

	Currently Smoke or Use Any Tobacco (*n* = 384)	
	% (95% CI)	APR (95% CI)
HIV status		
HIV-positive	4.4% (3.5–5.6%)	1.46 (1.09–1.96) ^1^
HIV-negative	2.4% (2.1–2.8%)	Ref
Age group		
15–24 years	0.9% (0.6–1.2%)	Ref
25–34 years	3.2% (2.6–3.9%)	3.01 (2.11–4.30) ^1^
35–49 years	4.9% (4.1–5.8%)	4.16 (2.85–6.07) ^1^
Residence		
Urban	2.5% (2.0–3.0%)	1.78 (1.20–2.63) ^1^
Rural	2.9% (2.4–3.4%)	Ref
Highest education level attended or completed		
No education	6.1% (4.6–8.0%)	1.79 (1.22–2.63) ^1^
Primary	3.1% (2.6–3.7%)	1.16 (0.87–1.55)
Secondary or higher	1.8% (1.4–2.2%)	Ref
Employment status		
Currently employed	3.5% (3.0–4.1%)	1.24 (0.95–1.62)
Not currently employed	2.0% (1.6–2.5%)	Ref
Wealth index		
Lowest	4.4% (3.5–5.5%)	3.87 (2.18–6.88) ^1^
Lower	2.7% (2.1–3.6%)	2.39 (1.36–4.20) ^1^
Middle	2.5% (1.8–3.3%)	1.83 (1.09–3.09) ^1^
Higher	2.6% (1.9–3.4%)	1.49 (0.92–2.40)
Highest	1.7% (1.2–2.4%) ^2^	Ref
Recent sexual activity		
Sexually active in last 4 weeks	3.3% (2.8–3.9%)	1.23 (0.95–1.61)
Not sexually active in last 4 weeks	2.0% (1.6–2.5%)	Ref
Problems accessing health care		
Yes	2.8% (2.3–3.3%)	Ref
No	2.6% (2.1–3.2%)	1.05 (0.81–1.36)
Pregnancy status		
Currently pregnant	2.0% (1.3–3.1%) ^2^	Ref
Not currently pregnant or not sure	2.8% (2.4–3.1%)	1.24 (0.81–1.90)

Abbreviations: CI—confidence interval; DHS—Demographic and Health Survey; PR—prevalence ratio; APR—adjusted prevalence ratio. ^1^ Statistically significant based on confidence interval for APR not including 1.0. ^2^ Estimate based on 25–49 unweighted cases.

**Table 4:** Prevalence and adjusted prevalence ratios (APRs) for HIV status and selected characteristics with tobacco smoking status, frequency of smoked tobacco use, and intensity of smoked tobacco use among men—2018 DHS, Zambia.

	Tobacco Smoking Status				Frequency of Smoked Tobacco Use ^1^				Intensity of Smoked Tobacco Use ^1^					
	Currently Smoke Tobacco (*n* = 2338)		Formerly Smoked Tobacco (*n* = 843)		Every Day (*n* = 1776)		Some Days (*n* = 562)		Five or More Times per Day (*n* = 902)		1–4 Times per Day (*n* = 856)		<1 Time per Day (*n* = 562)	
	% (95% CI)	APR (95% CI)	% (95% CI)	APR (95% CI)	% (95% CI)	APR (95% CI)	% (95% CI)	APR (95% CI)	% (95% CI)	APR (95% CI)	% (95% CI)	APR (95% CI)	% (95% CI)	APR (95% CI)
HIV status														
HIV-positive	27.8% (24.6–31.3%)	1.22 (1.08–1.37) ^2^	12.2% (9.85–15.0%)	1.12 (1.05–1.19) ^2^	76.8% (69.9–82.6%)	1.02 (0.94–1.11)	23.2% (17.4–30.1%)	0.94 (0.71–1.24)	40.8% (34.1–47.9%)	0.97 (0.80–1.16)	34.3% (28.1–41.0%)	1.01 (0.97–1.04)	25.0% (19.1–31.8%)	1.04 (0.84–1.30)
HIV-negative	18.7% (17.6–19.9%)	Ref	7.4% (6.7–8.2%)	Ref	75.6% (73.1–77.9%)	Ref	24.4% (22.1–26.9%)	Ref	39.3% (36.7–41.9%)	Ref	36.2% (33.5–39.0%)	Ref	24.6% (22.3–27.1%)	Ref
Age group														
15–24 years	8.0% (6.9–9.1%)	Ref	6.3% (5.4–7.4%)	Ref	63.7% (57.0–69.9%)	Ref	36.3% (30.1–43.0%)	Ref	26.2% (21.3–31.8%)	Ref	36.7% (30.6–43.3%)	Ref	37.1% (30.6–44.1%)	Ref
25–34 years	24.7% (22.6–26.9%)	2.17 (1.89–2.51) ^2^	8.3% (7.1–9.8%)	1.73 (1.56–1.92) ^2^	70.9% (67.0–74.6%)	1.07 (0.97–1.19)	29.1% (25.4–33.0%)	0.85 (0.68–1.06)	35.5% (31.5–39.8%)	1.33 (1.08–1.63) ^2^	34.8% (30.6–39.3%)	1.00 (0.98–1.03)	29.7% (26.1–33.5%)	0.76 (0.62–0.92) ^2^
35–49 years	28.5% (26.4–30.7%)	2.38 (2.06–2.75) ^2^	8.8% (7.6–10.1%)	1.82 (1.64–2.02) ^2^	81.9% (78.6–84.9%)	1.19 (1.09–1.31) ^2^	18.1% (15.2–21.4%)	0.60 (0.47–0.76) ^2^	45.6% (41.7–49.6%)	1.71 (1.40–2.09) ^2^	36.2% (32.5–40.1%)	0.93 (0.86–0.98) ^2^	18.2% (15.2–21.6%)	0.55 (0.44–0.67) ^2^
50–59 years	31.6% (28.3–35.1%)	2.76 (2.36–3.23) ^2^	10.7% (8.6–13.3%)	1.95 (1.76–2.17) ^2^	84.6% (79.5–88.6%)	1.21 (1.09–1.34) ^2^	15.4% (11.4–20.5%)	0.56 (0.40–0.79) ^2^	47.4% (40.2–54.6%)	1.75 (1.40–2.18) ^2^	37.1% (29.7–45.2%)	0.92 (0.86–1.00)	15.6% (11.5–20.7%)	0.53 (0.41–0.68) ^2^
Residence														
Urban	17.5% (15.8–19.5%)	1.63 (1.41–1.88) ^2^	9.0% (7.8–10.4%)	1.32 (1.22–1.42) ^2^	70.8% (66.1–75.1%)	1.02 (0.94–1.10)	29.2% (24.9–33.9%)	0.95 (0.74–1.22)	41.3% (37.1–45.6%)	1.08 (0.93–1.26)	28.7% (24.0–33.9%)	0.98 (0.94–1.02)	30.0% (25.8–34.6%)	0.91 (0.75–1.10)
Rural	21.1% (19.7–22.5%)	Ref	6.9% (6.0–8.0%)	Ref	78.9% (76.4–81.3%)	Ref	21.1% (18.7–23.6%)	Ref	38.2% (35.3–41.3%)	Ref	40.6% (37.8–43.5%)	Ref	21.2% (18.8–23.7%)	Ref
Highest education level attended or completed														
No education	27.6% (22.5–33.4%)	1.26 (1.04–1.52) ^2^	7.5% (5.0–11.0%) ^3^	1.15 (1.03–1.28) ^2^	- ^4^	- ^4^	- ^4^	- ^4^	- ^4^	- ^4^	- ^4^	- ^4^	- ^4^	- ^4^
Primary	25.0% (23.3–26.8%)	1.25 (1.13–1.39) ^2^	7.2% (6.3–8.3%)	1.15 (1.08–1.22) ^2^	81.9% (78.9–84.5%)	1.16 (1.08–1.25) ^2^	18.1% (15.5–21.1%)	0.64 (0.51–0.79) ^2^	41.7% (38.2–45.3%)	1.26 (1.09–1.46) ^2^	39.8% (36.5–43.3%)	0.95 (0.92–0.98) ^2^	18.5% (15.8–21.4%)	0.75 (0.63–0.90) ^2^
Secondary or higher	15.1% (13.7–16.7%)	Ref	8.2% (7.4–9.2%)	Ref	66.5% (62.5–70.3%)	Ref	33.5% (29.7–37.5%)	Ref	35.8% (32.2–39.6%)	Ref	30.3% (27.0–33.9%)	Ref	33.8% (30.1–37.9%)	Ref
Employment status														
Currently employed	22.7% (21.4–24.1%)	1.32 (1.16–1.50) ^2^	8.6% (7.8–9.5%)	1.19 (1.09–1.30) ^2^	76.2% (73.7–78.6%)	1.00 (0.92–1.09)	23.8% (21.5–26.3%)	0.99 (0.76–1.30)	39.2% (36.5–41.9%)	0.95 (0.79–1.14)	36.7% (34.1–39.5%)	1.01 (0.96–1.07)	24.1% (21.8–26.6%)	1.07 (0.84–1.36)
Not currently employed	9.7% (8.3–11.4%)	Ref	5.5% (4.3–6.9%)	Ref	72.3% (64.7–78.7%)	Ref	27.8% (21.3–35.3%)	Ref	41.3% (34.2–48.8%)	Ref	30.5% (23.4–38.7%)	Ref	28.2% (21.7–35.8%)	Ref
Wealth index														
Lowest	30.1% (27.9–32.4%)	2.62 (2.13–3.22) ^2^	6.5% (5.3–8.0%)	1.77 (1.55–2.02) ^2^	80.6% (76.7–84.0%)	1.04 (0.91–1.19)	19.4% (16.1–23.3%)	0.89 (0.59–1.34)	37.0% (32.8–41.4%)	0.94 (0.71–1.24)	43.5% (39.2–47.9%)	1.01 (0.94–1.09)	19.5% (16.1–23.4%)	1.08 (0.76–1.53)
Lower	23.8% (21.6–26.2%)	2.16 (1.76–2.64) ^2^	7.2% (5.7–8.9%)	1.63 (1.42–1.87) ^2^	82.6% (78.4–86.2%)	1.07 (0.95–1.21)	17.4% (13.8–21.6%)	0.79 (0.53–1.18)	41.6% (37.0–46.4%)	1.02 (0.78–1.34)	40.9% (36.5–45.5%)	0.99 (0.92–1.07)	17.5% (13.9–21.8%)	0.97 (0.68–1.37)
Middle	18.7% (16.6–20.9%)	1.66 (1.36–2.02) ^2^	6.9% (5.7–8.2%)	1.41 (1.23–1.62) ^2^	71.5% (66.2–76.3%)	0.96 (0.85–1.09)	28.5% (23.7–33.8%)	1.11 (0.78–1.57)	36.4% (31.9–41.3%)	0.88 (0.67–1.14)	35.0% (30.0–40.4%)	1.03 (0.96–1.10)	28.6% (23.8–33.9%)	1.17 (0.84–1.63)
Higher	17.0% (15.1–19.0%)	1.28 (1.07–1.54) ^2^	9.0% (7.3–11.0%)	1.20 (1.05–1.37) ^2^	71.6% (65.4–77.1%)	1.01 (0.91–1.12)	28.4% (22.9–34.6%)	0.97 (0.71–1.33)	42.1% (36.0–48.4%)	1.00 (0.77–1.31)	28.8% (22.6–35.8%)	1.00 (0.93–1.08)	29.2% (24.2–34.8%)	1.00 (0.70–1.41)
Highest	11.8% (10.0–13.9%)	Ref	8.9% (7.4–10.7%)	Ref	67.7% (59.4–75.0%)	Ref	32.3% (25.1–40.6%)	Ref	40.9% (32.8–49.6%)	Ref	25.6% (18.2–34.8%)	Ref	33.5% (25.9–42.1%)	Ref
Recent sexual activity														
Sexually active in last 4 weeks	24.1% (22.7–25.7%)	1.02 (0.93–1.12)	8.9% (8.0–9.9%)	1.01 (0.96–1.07)	77.3% (74.7–79.8%)	1.03 (0.96–1.10)	22.7% (20.2–25.4%)	0.92 (0.76–1.13)	39.8% (36.9–42.8%)	1.03 (0.90–1.18)	37.3% (34.3–40.4%)	0.99 (0.96–1.02)	22.9% (20.5–25.6%)	0.96 (0.81–1.14)
Not sexually active in last 4 weeks	13.0% (11.7–14.4%)	Ref	6.3% (5.4–7.3%)	Ref	71.5% (67.0–75.7%)	Ref	28.5% (24.3–33.1%)	Ref	38.5% (34.0–43.2%)	Ref	32.5% (28.2–37.0%)	Ref	29.0% (24.8–33.7%)	Ref
Current smokeless tobacco use														
Yes	70.4% (56.7–81.2%)	3.54 (2.95–4.25) ^2^	- ^4^	- ^4^	- ^4^	- ^4^	- ^4^	- ^4^	- ^4^	- ^4^	- ^4^	- ^4^	- ^4^	- ^4^
No	19.0% (17.9–20.2%)	Ref	7.8% (7.0–8.6%)	Ref	76.1% (73.6–78.4%)	Ref	23.9% (21.6–26.4%)	Ref	39.6% (37.1–42.2%)	Ref	36.2% (33.6–38.8%)	Ref	24.3% (22.0–26.7%)	Ref

Abbreviations: CI—confidence interval; DHS—Demographic and Health Survey; PR—prevalence ratio; APR—adjusted prevalence ratio. ^1^ Among current smokers only. ^2^ Statistically significant based on confidence interval for APR not including 1.0. ^3^ Estimate based on 25–49 unweighted cases. ^4^ Data suppressed because the estimate was based on fewer than 25 unweighted cases.

## Data Availability

Restrictions apply to the availability of these data. Data were obtained with permission from the Demographic and Health Surveys (DHS) Program. Available online: https://www.dhsprogram.com (accessed on 11 February 2022).

## Appendix A

Responses to the following questions were used to create tobacco-related variables for women: “Do you currently smoke cigarettes every day, some days, or not at all?” and “Do you currently smoke or use any other type of tobacco every day, some days, or not at all?”.

## Appendix B

Responses to the following questions were used to create tobacco-related variables for men: “Do you currently smoke tobacco every day, some days, or not at all?”, “In the past, have you ever smoked tobacco every day, some days, or not at all?”, and “On average, how many (manufactured cigarettes/hand-rolled cigarettes/pipes full of tobacco/cigars, cheroots, or cigarillos/number of water pipe (shisha) sessions/other types of tobacco) do you currently smoke each day?”.
